# Gene expression analysis of mammary tissue during fetal bud formation and growth in two pig breeds – indications of prenatal initiation of postnatal phenotypic differences

**DOI:** 10.1186/1471-213X-12-13

**Published:** 2012-04-26

**Authors:** Kunsuda Chomwisarutkun, Eduard Murani, Siriluck Ponsuksili, Klaus Wimmers

**Affiliations:** 1 Leibniz Institute for Farm Animal Biology, Research Unit `Molecular Biology´, Wilhelm-Stahl-Allee 2, 18196, Dummerstorf, Germany; 2 Leibniz Institute for Farm Animal Biology, Research Group `Functional Genome Analysis´, Dummerstorf, Germany

## Abstract

**Background:**

The mammary gland is key to all mammal species; in particular in multiparous species like pigs the number and the shape of functional mammary gland complexes are major determinants of fitness. Accordingly, we aimed to catalog the genes relevant to mammogenesis in pigs. Moreover, we aimed to address the hypothesis that the extent and timing of proliferation, differentiation, and maturation proccesses during prenatal development contribute to postnatal numerical, morphological and functional properties of the mammary gland. Thus we focused on differentially expressed genes and networks relevant to mammary complex development in two breeds that are subject to different selection pressure on number, shape and function of teats and show largely different prevalence of non-functional inverted teats. The expression patterns of fetal mammary complexes obtained at 63 and 91 days post conception (dpc) from German Landrace (GL) and Pietrain (PI) were analyzed by Affymetrix GeneChip Porcine Genome Arrays.

**Results:**

The expression of 11,731 probe sets was analysed between the two stages within and among breeds. The analysis showed the largest distinction of samples of the breed GL at 63 dpc from all other samples. According to Ingenuity Pathways Analysis transcripts with abundance at the four comparisons made (GL63-GL91, PI63-PI93, GL63-PI63 and GL91-PI91) were predominantly assigned to biofunctions relevant to `cell maintenance, proliferation, differentiation and replacement´, `organismal, organ and tissue development´ and `genetic information and nucleic acid processing´. Moreover, these transcripts almost exclusively belong to canonical pathways related to signaling rather than metabolic pathways. The accumulation of transcripts that are up-regulated in GL compared to PI indicate a higher proliferating activity in GL, whereas processes related to differentiation, maturation and maintenance of cells are more prominent in PI. Differential expression was validated by quantitative RT-PCR of five genes (*GAB1, MAPK9, PIK3C2B, PIK3C3 and PRKCH*) that are involved in several relevant signaling pathways.

**Conclusions:**

The results indicate that mammary complex development in PI precedes GL. The differential expression between the two breeds at fetal stages likely reflects the prenatal initiation of postnatal phenotypes concerning the number and shape as well as functionality of teats.

## Background

The development of the mammary gland is initiated during fetal stage. In the pig, the first visible structure at embryonic day 23 to 28 (E23 to E28) are elevated epidermal ridges or milk lines which are extending between forelimb to hindlimb on each side of trunk. The milk lines are a thickening of the ectoderm or the epidermis which are then fragment into individual buds. The formation of mammary placodes appears along each side of the body. In between E28 and E40, the placodes develop into bulb-shaped buds of epithelial cells by invagination into the underlying mesenchyme. Subsequently the size of the buds is slowly increasing and at E80 the mesenchymal cells surrounding the epithelial buds start to condense to become the mammary mesenchyme. Only late in prenatal development the epithelial buds elongate to the mammary mesenchyme to form a sprout, which creates a small duct. The sprout penetrates through fat pads. It starts to the ductal elongation and side branching about 10–15 times to form a rudimentary ductal tree. The mammary glands remain at this rudimentary stage, while the epithelial duct slowly grows until it reaches puberty [1-5].

Essentially, the development of mammary gland depends on growth hormones and growth factors. Moreover, the mammary gland development at fetal stages is apparently autonomous. The initiation of the mammary gland development and the early stage of morphogenesis are controlled by reciprocal interaction between epithelial and surrounding mesenchymal cells. The differentiation of mammary epithelia is also induced by the mammary mesenchyme [6-8]. During the differentiation at fetal stages the fate of cells towards their specialization as member of a population of cells typical for a tissue or organ is programmed. Accordingly, fetal development has implications on postnatal phenotypes. The mammary gland is key to all mammal species; in multiparous pigs the number and the shape of functional mammary gland complexes are major determinants of the mothering ability of sows.

In order to catalog genes relevant to mammogenesis in pigs, we analyzed the transcriptome of the mammary buds at the phase of formation and growth, i.e. at 63 day post conception (dpc) and 91 dpc, when epithelial and mesenchymal cell undergo proliferation and differentiation processes.

In order to address the hypothesis that balancing of proliferation and differentiation of epithelial and mesenchymal cells during prenatal development contributes to the postnatal shape and functionality of the mammary gland we compared fetal specimens obtained from two divergent breeds, German Landrace and Pietrain. Whereas in dam lines like German Landrace young sows are strongly selected for numerical, morphological and functional properties of the mammary gland, in sire lines like Pietrain these are not obligatory selection criterion. Accordingly, mammary complexes of both breeds differ in terms of number of teats and their distribution along the body and their symmetry at bold sides as well as the occurrence of additional non-functional teats including inverted teats. In fact, the examination of teat complexes of more than 2000 carcasses at the abattoir revealed mean teat numbers of around 15 and 13 in German Landrace and Pietrain, respectively [9], own observations]. The breeds also differ in the number of non-functional teats; interestingly, we observed different incidences of inverted teats of 12% in dam lines and 48% in Pietrain [10,11]. Thus differences of expression profiles of prenatal teat tissues between the two breeds could be indicative for molecular routes impertinent for the postnatal morphology and functionality of the mammary complexes.

The inverted teat defect is a polygenetic inherited liability trait marked by a decrease the number of functional teats thus causing animal welfare concerns due to increased piglet mortality and incidence of mastitis. It has been suggested that impaired prenatal development contributes to the emerging of inverted teats [10,12]. Moreover, we have previously demonstrated that genes of growth factor signaling pathways show differential expression depending on the teat phenotype and the affection status of the individual [11,13]. We aimed to elucidate whether molecular routes found to be affected due to the development of inverted teats at postnatal stages may already play a role during prenatal development. However, at prenatal stages the phenotype of inverted teat is not yet visible and the development of the teats cannot be predicted. In general, beside clones of animals that express a certain trait that is only visible during postnatal life but that is initiated by prenatal events, fetal samples of breeds that largely differ in that trait are the best available model.

The study was conducted with commercial genome-wide microarray (Affymetrix GeneChip Porcine Genome Array) to identify the differentially expressed genes and networks relevant to the development of mammary complexes in the two breeds and contributes to the understanding of molecular routes relevant to postnatal emergence of differences in morphological and functional properties.

## Results

### Gene expression

**Figure 1:**
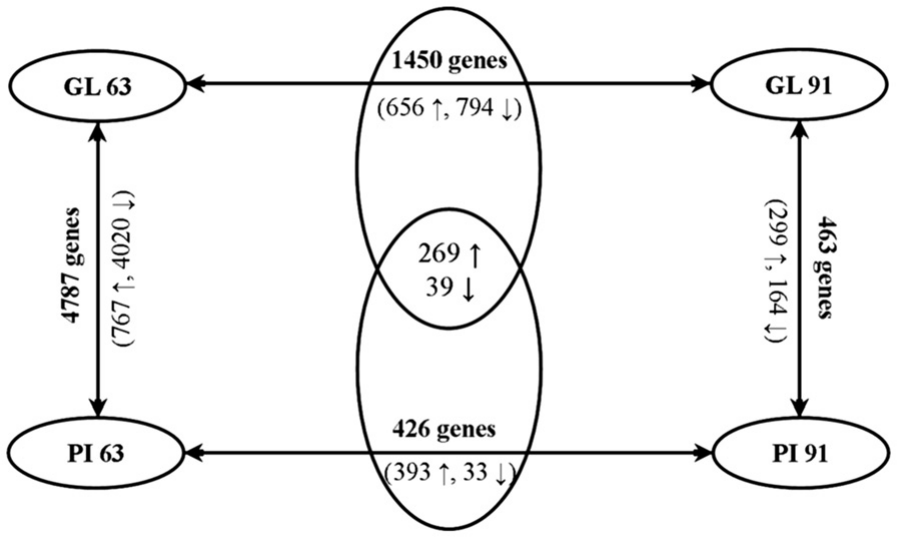
**The number of significant differentially expressed genes in same breeds or stages and different breeds or stages.** in bold: numbers of transcripts with difference abundance at the respective comparisons (GL63-GL91, PI63-PI93, GL63-PI63 and GL91-PI91) at p < 0.05. in parenthesis: number of transcripts with higher or lower abundance relative to the breed PI (↑ = PI higher or ↓ = PI lower)during development from 63 dpc to 91 dpc (↑ = increase, ↓ = decrease).

In order to identify differential gene expression in mammary gland development depending on stage (63 dpc and 91 dpc) and breed (GL and PI) probe sets (subsequently also referred to as genes or transcripts) with present calls in at least 50% of the samples were selected for statistical analysis summing up to 11,731 probe sets. Differential expression was evaluated between breeds within stages. Further we listed differentially expressed genes (DE-genes) between stages within breed. These two lists of DE-genes were compared. The commonly temporally regulated genes were considered separate from those genes that were specific to either of the two breeds because only the later reflect breed differences, whereas commonly regulated genes between stages in both breeds are not likely to contribute to the initiation of divergence of the postnatal phenotype among the breeds. The numbers of significant differentially expressed genes are summarized in Figure 1. Fold changes (FC) varied between 1.2 and 50.2. Most pronounced differences were found between breeds at stage 63 dpc: at p < 0.05 corresponding to q = 0.005-0.03 there were 4787 DE-genes with median FC of 2.1 and a maximum FC of 50.2. Also between stages within the breed GL considerable transcriptomic differences were obvious (number of DE-genes: 1758 at p < 0.05 corresponding to q = 0.001-0.14; FC_median_ = 1.7; FC_max_ = 19.2; number of DE-genes temporally regulated but specific to GL: 1450 at p < 0.05 corresponding to q = 0.001-0.14; FC_median_ = 1.8; FC_max_ = 11.4), whereas between stages 63 dpc and 91 dpc in PI (number of DE-genes: 734 at p < 0.05 corresponding to q = 0.04-0.8; FC_median_ = 1.6; FC_max_ = 25.4; number of DE-genes temporally regulated but specific to PI: 426 genes at p < 0.05 corresponding to q = 0.04-0.8; FC_median_ = 1.7; FC_max_ = 19.7) and among GL and PI at 91 dpc (number of DE-genes: 463 at p < 0.05 corresponding to q = 0.5; FC_median_ = 1.7; FC_max_ = 11.9) the distinctness of the expression profiles was moderate.

### Ingenuity pathway analysis

**Figure 2:**
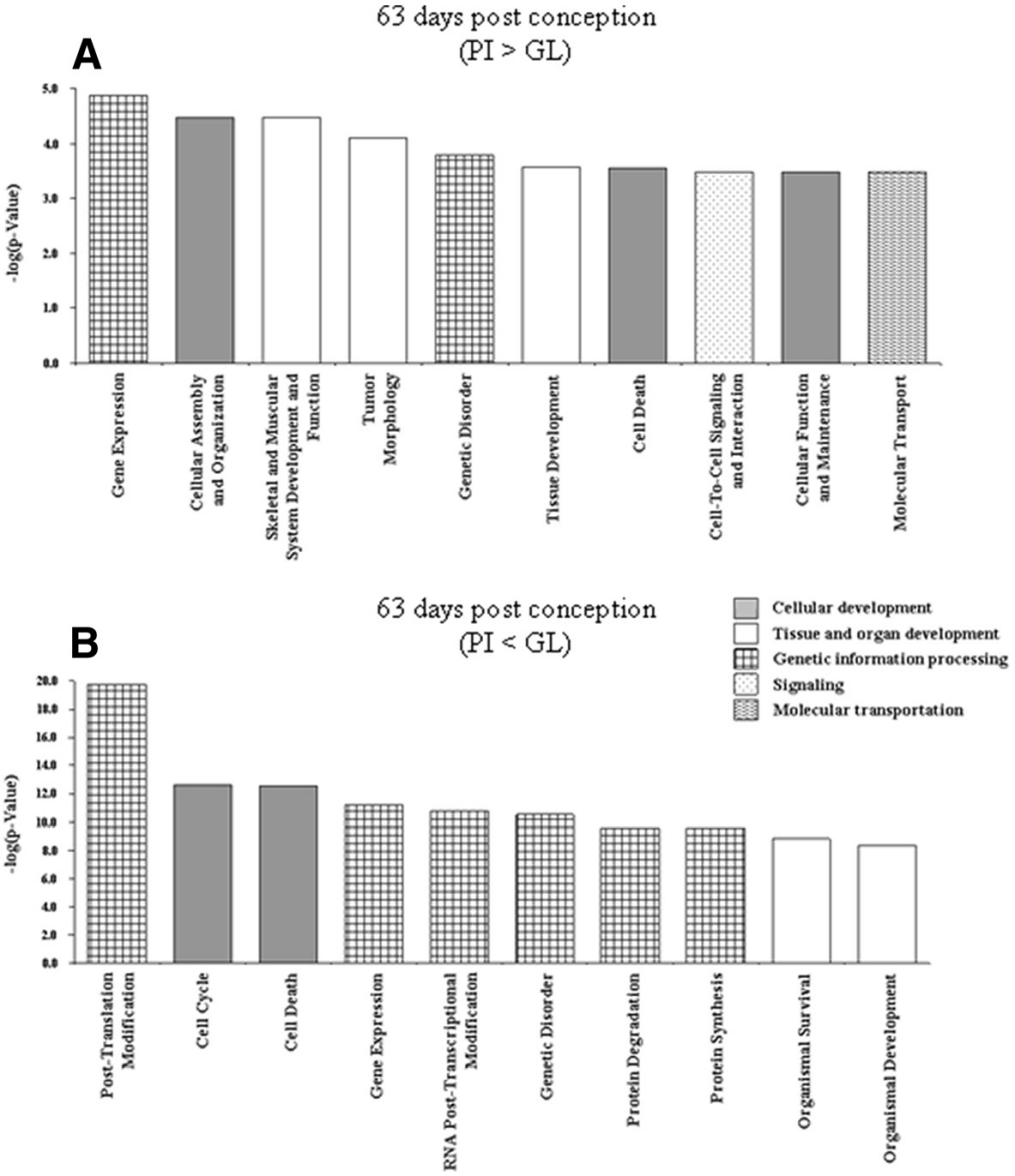
**Significant biofunctions (top ten according to p-value) representing genes differentially expressed between samples of mammary complexes of Pietrain and German Landrace at 63 dpc.(A)** PI > GL **(B)** PI < GL. All assignments significant after Benjamini–Hochberg correction, except `tissue development´, `cell death´, cell-to-cell signaling´, `cellular function and maintenance´, and `molecular transport´ in **(A).**

**Figure 3:**
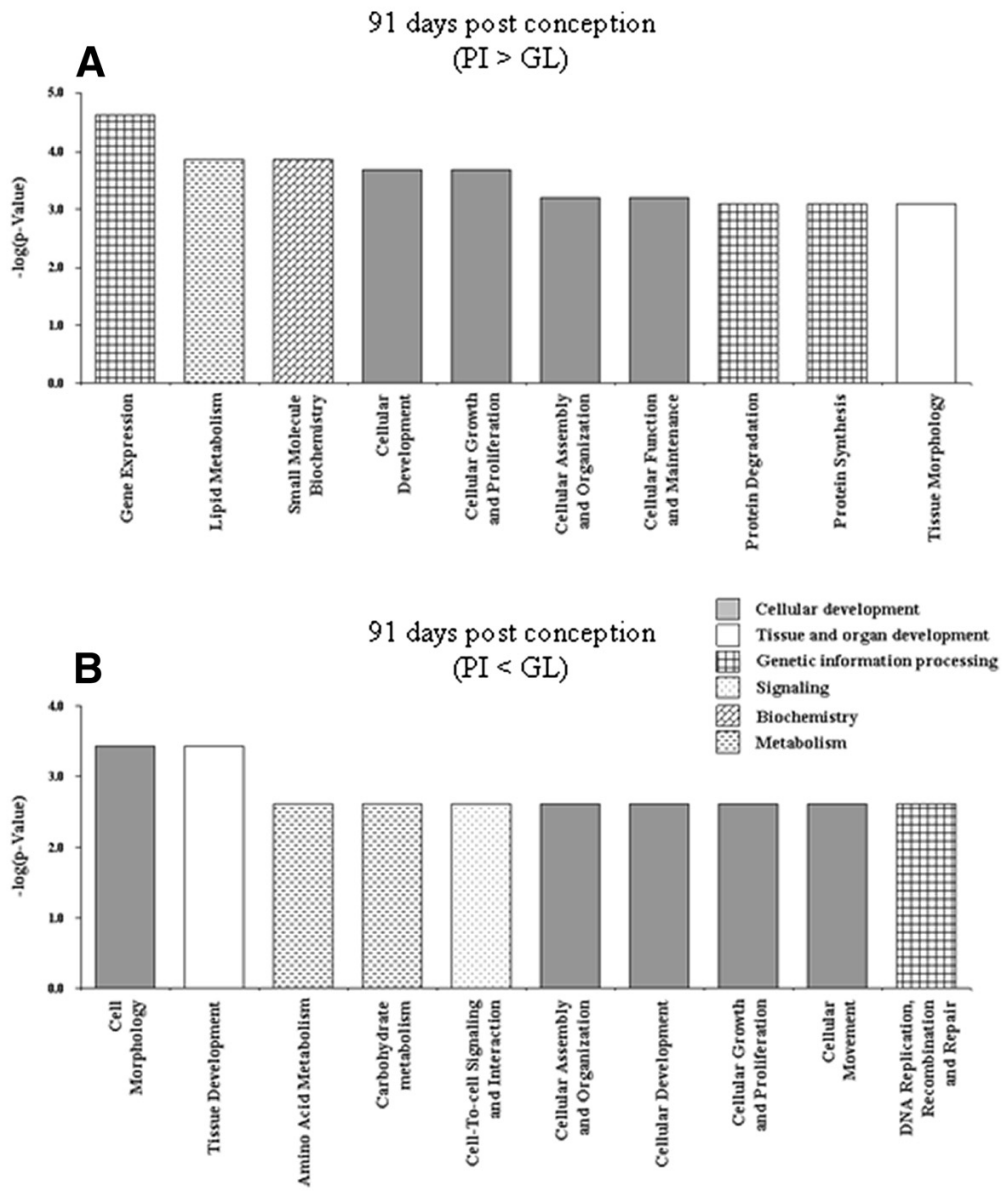
**Significant biofunctions (top ten according to p-value) representing genes differentially expressed between samples of mammary complexes of Pietrain and German Landrace at 91 dpc.(A)** PI > GL **(B)** PI < GL. All assignments significant after Benjamini–Hochberg correction.

**Figure 4:**
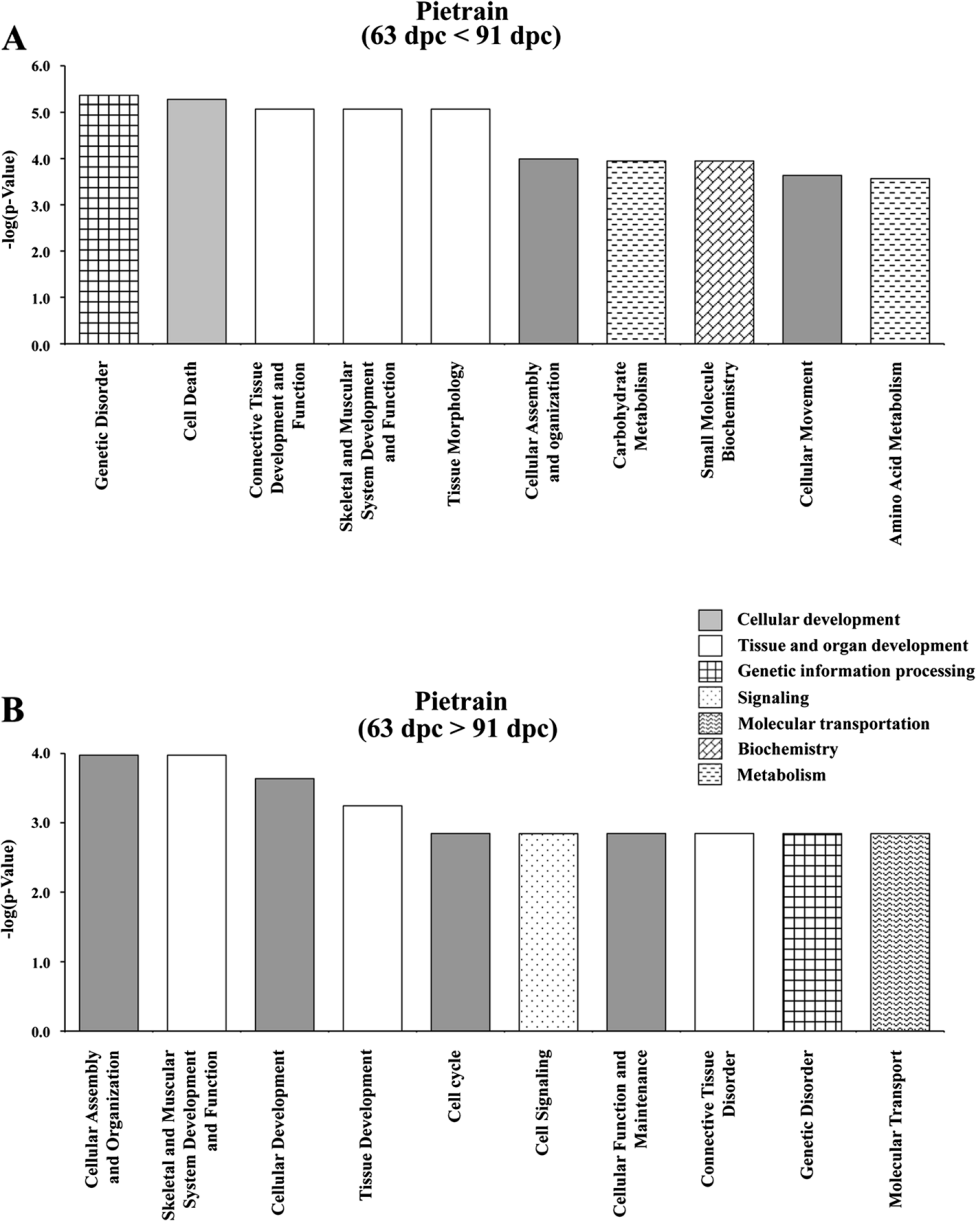
**Significant biofunctions (top ten according to p-value) representing genes differentially expressed in Pietrain at 63 dpc compared to 91 dpc.(A)** 63 dpc < 91 dpc **(B)** 63 dpc > 91 dpc. All assignments significant after Benjamini–Hochberg correction.

**Figure 5:**
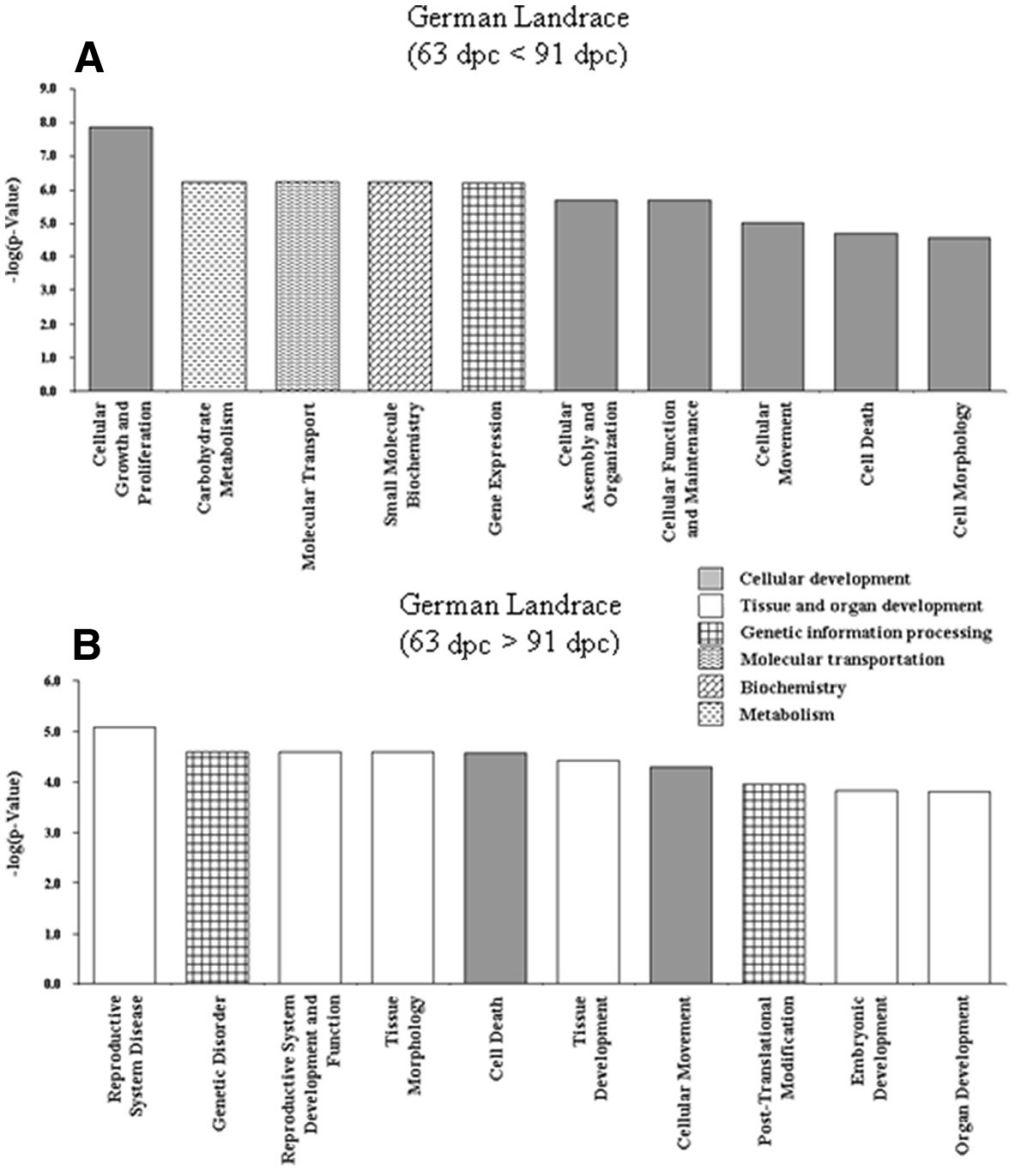
**Significant biofunctions (top ten according to p-value) representing genes differentially expressed in German Landrace at 63 dpc compared to 91 dpc.(A)** 63 dpc < 91 dpc **(B)** 63 dpc > 91 dpc. All assignments significant after Benjamini–Hochberg correction.

Four lists of DE-genes were imported to the Ingenuity Pathways Analysis (IPA) in order to assign them to groups or categories of biofunctions and to canonical pathways as defined in the Ingenuity Knowledge Base (Genes) and to test for significant enrichment of DE-genes within these groups. The DE-genes were predominantly assigned to biofunctions relevant to `cell maintenance, proliferation, differentiation and replacement´, `organismal, organ and tissue development´ and `genetic information and nucleic acid processing´. Referring to the most significant functions named by IPA within the IPA-categories listed in Figures 2, 3, 4, 5 elucidates the developmental status of the mammary gland tissues within the four groups compared here. Those genes that are higher expressed in PI than in GL at 63 dpc were assigned to genetic information processing, in particular transcription and transactivation (IPA-category: `gene expression´), cellular processes including formation of filaments and intercellular junction (IPA-category: `cellular assembly and organization´), survival of cells (IPA-category: `cell death), and cytostasis (IPA-category: `cellular function and maintenance´)(Figure 2A). At 63 dpc in DL many genes annotated to biofunctions related to genetic information and nucleic acid processing are higher expressed than in PI; this covers functions of transcription (IPA-category: `gene expression´), RNA processing and splicing (IPA-category: `RNA post-transcriptional modification´), apoptosis (IPA-category: `cell death´) but also cell division (IPA-category: `cell cycle´) and also includes genes relevant to early onset of breast cancer (IPA-category: `genetic disorder´) (Figure 2B). The accumulation of DE-genes that are up-regulated in DL compared to PI indicate a higher proliferating activity in DL, whereas processes related to maintenance and differentiation and maturation of cells are more prominent in PI at 63 dpc.

At 91 dpc biofunctions related to cellular development are predominantly regulated in both breeds (Figure 3A, B). In GL genes with function in cell differentiation including the processes involved in commitment of a cell to a specific fate and its subsequent development to the mature state (IPA-category: `cellular development´) as well as stem cell proliferation (IPA-category: `cellular growth and proliferation´) are up-regulated compared to PI (Figure 3B). Cell maturation, i.e. developmental processes, independent of morphogenetic (shape) change, that are required for a cell to attain its fully functional state (IPA-category: `cellular development´) and growth (IPA-category: `cellular growth and proliferation´) are biofunctions covering a significant number of genes higher expressed in PI than in GL at 91 dpc (Figure 3A). Thus also at 91 dpc the results suggest that the tissue of the mammary complex has already reached a higher degree of maturity in PI than GL. However, none of the transcripts considered here in this comparison among breeds at 91 dpc reached a false discovery rate below 0.5.

Corresponding to the differential regulation between breeds within stages the temporal regulation between 63 dpc and 91 dpc that is limited to PI indicates a high expression of genes related to differentiation and maintenance of cell, i.e. survival of cells (IPA-category: `cell death´), organization of organelles and cytoskeleton (IPA-category: `cellular assembly and organisation´) and remodelling of tissue and quantity of connective tissue cells (IPA-categories: `connective tissue development and function´ as well as `tissue morphology´) (Figure 4). When only taking into account transcripts with different abundance at p < 0.001 (corresponding to q < 0.27) 44 remain that do not provide a meaningful IPA. The temporally regulated genes that are restricted to the breed GL were assigned to processes impertinent to dynamic changes of the cell population within the tissue at 63 dpc with functions like proliferation of cells (IPA-category: `cellular growth and proliferation´), transcription (IPA-category: `gene expression´), apoptosis (IPA-category: `cell death´), and cell migration (IPA-category: `cellular movement´) (Figure 5). Assignment to biofunctions of commonly regulated genes between stages in both breeds is shown in the supplementary material (Additional file 1: Figure S1).

**Figure 6:**
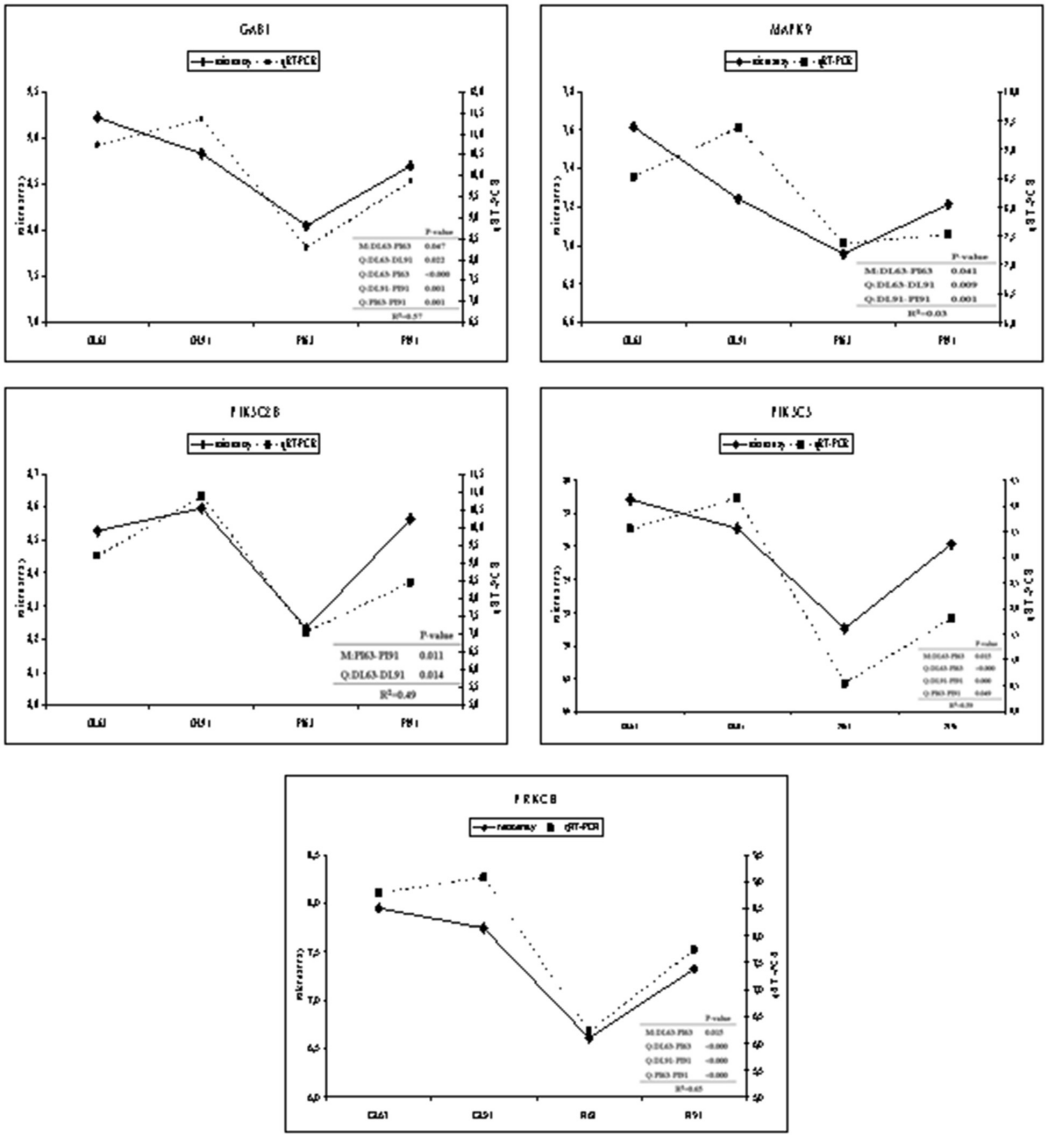
**Comparison of microarray (left y-axis) and quantitative real time PCR (right y-axis) data of five genes.** Graphs show log(2) mean values of transcript abundance of the breed categories `German Landrace´ (GL), and `Pietrain´ (PI) at the prenatal stage of 63 and 91 day post conception `GL63´, `GL91´, `PI63´ and `PI91´.

IPA towards assignment of DE-genes to canonical pathways pointed to signaling pathways important for cellular proliferation, differentiation, development and growth as detailed in Tables 1 and 2. We selected five genes (*GAB1, MAPK9, PIK3C2B, PIK3C3 and PRKCH*) that are involved in several signaling pathways and therefore were redundantly listed (in bold in Tables 1 and 2) for examination of their expression by qRT-PCR (Figure 6). In general, the breed and stage dependent differences of relative transcript abundance as found by microarray analysis and qRT-PCR were in good agreement (Figure 6). The relative transcript abundance according to microarrays and qRT-PCR were significantly correlated at R² = 0.49 - 0.65, except for MAPK9. For MAPK9 microarrays indicated highest expression GL at 63 dpc, whereas qRT-PCR indicated an even higher expression in GL at 91 dpc; however in both analyses expression in PI was lower than in GL at any of the two stages.

**Table 1:** Assignment of temporally regulated DE-genes to canonical pathways in German Landrace and Pietrain, respectively

**Ingenuity Canonical Pathways**	**63 dpc vs. 91 dpc in GL or PI**	**p-value^1^**	**Ratio^2^**	**Molecules**
Angiopoietin Signaling	GL	0.008	0.143	STAT5A, PAK4, AKT2, IKBKG, TNIP1, GRB14, ANGPT1, PAK6, PTPN11, MRAS, IKBKAP
	PI	0.065	0.065	**PIK3C2B**, PAK4, IKBKG, TNIP1, PIK3R4
Chemokine Signaling	GL	0.028	0.133	GNAI3, PLCB4, JUN, MYL2, CXCL12, MRAS, GNAQ, PPP1R12A, MAPK12, CAMK2G
	PI	0.393	0.040	CAMK2A, MYL2, CAMK2G
ILK Signaling	GL	0.005	0.120	MYH10, AKT2, VEGFB, MYL2, TNFRSF1A, CREB3, MYH11, MAPK12, MYL9, NCK2, RHOQ, JUN, RHOG, IRS1, RHOU, CHD1, MYH9, LEF1, ACTG2, PTGS2, PPP2R5E, ACTC1, ACTN1
	PI	0.002	0.073	**PIK3C2B**, MYL2, TNFRSF1A, VEGFC, MYH11, PIK3R4, MYL6B, PPP2R5A, ITGB3, PPP2R4, PPP2R2B, MYH3, PTGS2, ACTC1
Integrin Signaling	GL	0.011	0.112	CAPN5, FYN, AKT2, PAK4, ARHGAP26, TSPAN7, MYL2, PAK6, ARHGEF7, TNK2, MYLK, NCK2, RHOG, RHOQ, MRAS, RHOU, PPP1R12A, ITGA1, ACTG2, ACTC1, ACTN1, RAP2A, RAPGEF1
	PI	0.275	0.039	**PIK3C2B**, PAK4, MYL2, TSPAN7, PIK3R4, ACTC1, TTN, ITGB3
TGF-β Signaling	GL	0.008	0.145	SMAD2, BMPR1B, JUN, SMAD9, TGFB1, MRAS, TGFB3, TGFB2, SMAD6, PITX2, SMAD1, INHBB
	PI	0.228	0.048	SMAD2, TGFB1, TGFB3, INHBB
Wnt/β-catenin Signaling	GL	0.019	0.114	AKT2, SFRP2, APPL2, PPARD, WNT2B, MARK2, GNAQ, APPL1, CDH2, JUN, TGFB1, CSNK2A1, TGFB2, TGFB3, NR5A2, FZD5, LEF1, SFRP1, PPP2R5E, TCF7L2
	PI	0.046	0.057	CDH2, CDH1, PPP2R4, TGFB1, PPP2R2B, CD44, TGFB3, FZD6, TLE1, PPP2R5A
Cyclins and Cell Cycle Regulation	GL	0.023	0.124	CCND3, PA2G4, HDAC8, TGFB1, E2F1, HDAC7, E2F5, TGFB3, TGFB2, PPP2R5E, SKP2
	PI	0.003	0.090	CCNE2, PPP2R4, TGFB1, PPP2R2B, TGFB3, CCNB2, ATR, PPP2R5A
Aryl Hydrocarbon Receptor Signaling	GL	0.007	0.116	ALDH4A1, NFIC, MED1, HSPB2, CYP1B1, CTSD, ALDH1A1, JUN, CCND3, NCOA2, TGFB1, E2F1, TGFB3, TGFB2, IL1B, NFIB, NCOR2, ESR1
	PI	0.004	0.071	ALDH4A1, CCNE2, ALDH1A1, TGFB1, TGFB3, IL1B, ALDH18A1, NCOR2, ATR, ESR1, NCOA3
Gα12/13 Signaling	GL	0.043	0.109	F2RL2, AKT2, F2R, MYL2, MAPK12, LPAR3, CDH11, MYL9, CDH2, IKBKG, JUN, LPAR1, MRAS, MAPK7
	PI	0.005	0.078	**PIK3C2B**, CDH2, CDH1, IKBKG, MYL2, MEF2C, MAP3K5, MAPK7, PIK3R4, MYL6B
Glucocorticoid Receptor Signaling	GL	0.025	0.095	ICAM1, GTF2A2, TSC22D3, IKBKG, JUN, NCOA2, TGFB1, MRAS, TGFB2, GTF2H5, POLR2H, NCOR1, FKBP5, TAF12, SMAD2, STAT5A, AKT2, MED1, TAF15, CEBPB, MAPK12, NCOA1, TGFB3, IL1B, PTGS2, NCOR2,ESR1
	PI	0.033	0.049	SMAD2, **PIK3C2B**, SMARCD2, CEBPB, PIK3R4, NCOA3, TSC22D3, IKBKG, TGFB1, TGFB3, IL1B, NCOR2, PTGS2, ESR1
Ceramide Signaling	GL	0.352	0.078	CTSD, AKT2, JUN, TNFRSF1A, MRAS, PPP2R5E, TNFRSF1B
	PI	0.044	0.067	**PIK3C2B**, PPP2R4, TNFRSF1A, PPP2R2B, PIK3R4, PPP2R5A
PPARα/RXRα Activation	GL	0.002	0.123	PPARA, SMAD2, MED1, ADCY3, GNAQ, ADCY6, MAP4K4, CAND1, PLCD1, PLCD3, IKBKG, PLCB4, JUN, TGFB1, FASN, IRS1, MRAS, TGFB3, TGFB2, IL1B, NCOR1, NCOR2, INSR
	PI	0.020	0.059	PLCD1, SMAD2, IKBKG, TGFB1, GNA11, ADCY6, TGFB3, IL1B, NCOR2, NCOA3, ABCA1
RhoA Signaling	GL	0.019	0.127	MYL2, RDX, WASF1, DLC1, LPAR3, MYLK, MYL9, LPAR1, IGF1R, PPP1R12A, CDC42EP1, ACTG2, ACTC1, PI4KA
	PI	0.007	0.082	RHPN2, IGF1, MYL2, EPHA1, IGF1R, ARHGAP12, MYL6B, ACTC1, TTN
Tight Junction Signaling	GL	0.004	0.127	MYH10, F2RL2, TIAM1, AKT2, MYL2, TNFRSF1A, PVRL3, MARK2, MYH11, CASK, MYL9, MYLK, JUN, TGFB1, TGFB3, TGFB2, MYH9, ACTG2, PPP2R5E, TNFRSF1B, ACTC1
	PI	0.005	0.072	MYL2, CLDN8, PPP2R4, TGFB1, TNFRSF1A, PPP2R2B, MYH3, TGFB3, MYH11, MYL6B, ACTC1, PPP2R5A
Estrogen-Dependent Breast Cancer Signaling	GL	0.076	0.110	STAT5A, AKT2, JUN, CREB3, IGF1R, MRAS, HSD17B7, ESR1
	PI	0.014	0.082	**PIK3C2B**, IGF1, IGF1R, PIK3R4, ESR1, HSD17B8
Mitotic Roles of Polo-Like Kinase	GL	0.424	0.079	CDC25B, TGFB1, CDC23, PPP2R5E, ANAPC13
	PI	0.012	0.095	PPP2R4, TGFB1, PPP2R2B, CCNB2, CDC27, PPP2R5A
PPAR Signaling	GL	0.000	0.170	PPARA, STAT5A, TNFRSF1A, PPARD, MED1, MAP4K4, IKBKG, JUN, NCOA1, MRAS, IL1B, NCOR1, PTGS2, NCOR2, INSR, TNFRSF1B, CITED2, PDGFRB
	PI	0.081	0.057	IKBKG, TNFRSF1A, IL1B, NCOR2, PTGS2, PDGFRB
Role of Tissue Factor in Cancer	GL	0.164	0.095	FYN, STAT5A, YES1, AKT2, PTPN11, MRAS, GNAQ, IL1B, GNA14, RPS6KA1, MAPK12
	PI	0.009	0.078	**PIK3C2B**, CTGF, CSF1, GNA11, VEGFC, IL1B, RPS6KA1, PIK3R4, ITGB3
TR/RXR Activation	GL	0.010	0.130	AKT2, NXPH2, MED1, THRA, KLF9, SCARB1, COL6A3, NCOA2, FASN, NCOA1, STRBP, NCOR1, NCOR2
	PI	0.063	0.060	KLF9, **PIK3C2B**, COL6A3, NCOR2, PIK3R4, NCOA3

^1^pathways are shown that were significant at p < 0.05 according to Fishers exact test in at minimum one of the three types of comparisons.

^2^ratio of number of differentially expression genes assigned to the pathway and the total number of genes assigned to the pathway in the Ingenuity Knowledge Base.

**Table 2:** Assignment of DE-genes to canonical pathways in the comparison between breeds at either 63 dpc or 91 dpc

**Ingenuity Canonical Pathways**	**PI vs. GL at 63 dpc or 91 dpc**	**p-value^1^**	**Ratio^2^**	**Molecules**
Clathrin-mediated Endocytosis Signaling	63	0.011	0.222	EPS15, STON2, CDC42, FGF2, ARPC5, NUMB, SH3GL2, ITGB8, PIK3R4, CD2AP, ACTR3, WASL, SNX9, IGF1, **PIK3C3**, ARPC3, DAB2, STAM, AAK1, PPP3CA, ACTA1, ATM, ITGB1, MYO6, ACTR2, PIK3C2A, SH3GL3, ACTB, CLTC, RAB7A, MET, CBL, SYNJ1, ARPC2, RAB11A, TFRC, UBC, PDGFD
	91	0.106	0.029	EPS15, FGF2, TFRC, DAB2, AAK1
Corticotropin Releasing Hormone Signaling	63	0.033	0.204	RAP1B, PRKACB, RAF1, MAPK1, ARPC5, CREB5, PRKAG1, PRKD3, PRKCA, null, ITPR2, CNR1, PTCH1, GNAQ, GNAI1, ADCY6, MAPK12, RAP1A, ATF2, GNAS, GNAI3, PRKCI, MAPK14, PRKAR2B, PRKAG2, **PRKCH**, GLI1, PRKCB
	91	0.258	0.022	PRKAR2B, ADCY3, PTGS2
Integrin Signaling	63	0.000	0.259	MAP2K4, RAP2B, RAF1, MYL2, MAPK1, ARPC5, ITGA8, KRAS, PIK3R4, PTEN, TSPAN3, RHOG, ARF4, CAV1, ITGAV, GSK3B, ACTA1, ATM, CAPN5, ACTR2, BCAR3, RAP1A, TTN, RHOQ, RND3, ARPC2, PPP1R12A, CAPN7, TSPAN6, RAP1B, FYN, PPP1CC, RALA, CDC42, PPP1CB, ITGB8, SHC1, ACTR3, WASL, RHOT1, **PIK3C3**, SOS1, ARPC3, ITGB1, PAK2, PIK3C2A, ASAP1, ACTB, ITGA2, MAPK8, ROCK1, WIPF1, ITGAX
		91	0.191	0.024	RALA, ASAP1, ARF4, ITGA8, TTN
PI3K/AKT Signaling	63	0.048	0.197	RAF1, MAPK1, INPPL1, KRAS, JAK2, MAP3K5, EIF4E, PTEN, BCL2, SHC1, IKBKG, SOS1, TSC2, GSK3B, MCL1, ITGB1, RPS6KB1, YWHAG, PPP2R5C, ITGA2, TYK2, YWHAZ, PPP2R5A, PPP2CB, **GAB1**, CDKN1B, PPP2R5E, PPP2R1B	
	91	0.296	0.021	PTGS2, PPP2R5A, BCL2	
α-Adrenergic Signaling	63	0.012	0.236	PRKACB, RAF1, MAPK1, GNB5, KRAS, PRKAG1, PHKA2, GNB1, GNB4, PHKB, PRKD3, PRKCA, null, ITPR2, GNAI1, ADCY6, GNAQ, GNAS, GNAI3, PRKCI, PRKAR2B, PRKAG2, **PRKCH**, GNG2, PRKCB	
	91	0.404	0.019	PRKAR2B, ADCY3	
IL-15 Signaling	63	0.047	0.229	STAT5A, RAF1, PIK3C2A, MAPK1, TYK2, KRAS, JAK2, AXL, MAPK12, PIK3R4, BCL2, SHC1, MAPK14, **PIK3C3**, SYK, ATM	
	91	0.267	0.029	STAT6, BCL2	
Myc Mediated Apoptosis Signaling	63	0.018	0.266	MAP2K4, YWHAG, PIK3C2A, MAPK8, YWHAZ, **MAPK9**, KRAS, PIK3R4, MAPK12, BCL2, SHC1, IGF1, **PIK3C3**, SOS1, CYCS, BID, ATM	
	91	0.255	0.031	APAF1, BCL2	
Protein Kinase A Signaling	63	0.000	0.228	PRKACB, MYH10, RAF1, TGFBR1, MAPK1, MYL2, PDE12, GNB5, AKAP3, CREB5, PPP1R14B, TGFBR2, GNB1, GNB4, PHKB, CAMK2A, TDP2, GSK3B, PRKD3, null, YWHAG, ITPR2, PTCH1, CREBBP, YWHAZ, RAP1A, MYL6B, TTN, ATF2, MYL9, AKAP13, ANAPC4, ANAPC5, PPP1R12A, **PRKCH**, LEF1, GNG2, PDE6D, AKAP12, RAP1B, PPP1CC, FLNB, PDE7A, AKAP8, PDIA3, PPP1CB, H3F3A/H3F3B, CDC23, PRKAG1, PHKA2, NFAT5, TGFB2, SMAD4, PPP3CA, PRKCA, AKAP5, ATF1, MAP3K1, ADCY6, GNAI1, GNAQ, ANAPC13, ROCK1, GNAS, GNAI3, PPP1R3D, PRKAR2B, PRKCI, ADD3, KDELR2, PRKAG2, AKAP9, PRKCB, ANAPC1	
	91	0.348	0.019	ADD3, PRKAR2B, ADCY3, AKAP3, AKAP7, TTN	
Molecular Mechanisms of Cancer	63	0.000	0.275	RAP2B,RAF1,TGFBR1,APH1B,TAB2,ARHGEF1,KRAS,RBL1,RB1,CAMK2A,HIPK2,PRKD3,ATM,SMAD2,TFDP1,PTCH1,CREBBP,RAP1A,CDH1,**GAB1**,E2F1,CYCS,CFLAR,RAP1B,FYN,RALA,CDC42,LRP6,BMPR2,CRK,JAK2,MAP3K5,GNA14,CHEK1,CASP6,**PIK3C3**,SOS1,E2F5,BID,BMP1,PAK2,GNAQ,ADCY6,MAPK8,GNAI3,RBPJ,ATR,BIRC2,PSEN1,PRKCB,MAP2K4,PRKACB,MAPK1,PIK3R4,TGFBR2,RHOG,GSK3B,RASA1,BIRC3,TYK2,CDK6,MAPK12,RALBP1,APC,RHOQ,CBL,PTPN11,RND3,FZD6,**PRKCH**,LEF1,FZD5,CDK2,HIF1A,E2F3,PRKAG1,BCL2,CDC25B,SHC1,FANCD2,RHOT1,BMPR1A,MAP3K7,TGFB2,SMAD4,PRKCA,ARHGEF12,PIK3C2A,HAT1,GNAI1,**MAPK9**,XIAP,GNAS,PRKCI,MAPK14,PRKAR2B,FZD4,NF1,BMP8B,PRKAG2,CDKN1B,GLI1,BCL2L11	
	91	0.017	0.029	PRKAR2B,RALA,FZD4,ADCY3,APAF1,RAPGEF3,E2F3,CASP7,E2F2,WNT5A,BCL2	
p53 Signaling	63	0.000	0.293	GADD45G, PIK3R4, PTEN, CHEK1, BCL2, RB1, CASP6, GADD45A, **PIK3C3**, GSK3B, HIPK2, ATM, TP53INP1, TP63, PIK3C2A, TOPBP1, MED1, THBS1, HDAC1, PERP, MAPK8, TP53BP2, KAT2B, PCNA, MAPK14, E2F1, ATR, CDK2, SIRT1	
	91	0.192	0.030	MED1, APAF1, BCL2	
VDR/RXR Activation	63	0.003	0.284	CYP24A1, SPP1, CCNC, MED1, IGFBP5, CEBPB, THBD, KLF4, NCOA3, GTF2B, PRKCI, SP1, GADD45A, NCOA2, MXD1, NCOA1, IGFBP3, TGFB2, **PRKCH**, CDKN1B, PRKD3, PRKCA, PRKCB	
	91	0.124	0.037	CYP24A1, MED1, MXD1	
Breast Cancer Regulation by Stathmin1	63	0.000	0.248	PRKACB, RAF1, CAMK1D, MAPK1, GNB5, KRAS, ARHGEF1, PIK3R4, PPP1R14B, GNB1, GNB4, CAMK2A, PRKD3, ATM, null, ITPR2, PPP2CB, E2F1, PPP1R12A, **PRKCH**, GNG2, CDK2, PPP1CC, CDC42, PPP1CB, E2F3, PRKAG1, SHC1, **PIK3C3**, SOS1, RB1CC1, E2F5, PRKCA, ARHGEF12, PIK3C2A, PPP2R5C, GNAI1, TUBA4A, ADCY6, GNAQ, PPP2R5A, ROCK1, GNAS, GNAI3, PPP1R3D, PRKCI, PRKAR2B, PRKAG2, CDKN1B, PPP2R5E, PPP2R1B, PRKCB	
	91	0.191	0.024	PRKAR2B, ADCY3, E2F3, E2F2, PPP2R5A	
ERK/MAPK Signaling	63	0.001	0.230	RAP1B, PRKACB, FYN, PPP1CC, RAF1, MAPK1, HSPB2, H3F3A/H3F3B, ETS2, PPP1CB, KRAS, CRK, PIK3R4, CREB5, PPP1R14B, EIF4E, PRKAG1, SHC1, **PIK3C3**, SOS1, MKNK1, PRKCA, ATM, ITGB1, MYCN, PAK2, YWHAG, PPP2R5C, ATF1, PIK3C2A, ITGA2, YWHAZ, MAPKAPK5, RAP1A, PPP2R5A, ATF2, PLA2G4A, PPP2CB, PPP1R3D, PRKCI, PRKAR2B, PRKAG2, PPP1R12A, PPP2R5E, PPP2R1B, ELK3, PRKCB	
	91	0.556	0.015	PRKAR2B, RAPGEF3, PPP2R5A	
RAR Activation	63	0.001	0.246	MAP2K4, PRKACB, NSD1, MAPK1, MAP3K5, JAK2,RBP1, PRKAG1, PTEN, PNRC1, TGFB2, SMAD4, GTF2H5, NR2F6, RDH13, PRKD3, CITED2, PRKCA, STAT5A, SMAD2, SRA1, PRMT2, IL3RA, RDH14, MED1, RDH11, MAP3K1, CREBBP, MAPK8, ADCY6, **MAPK9**, MAPK12, CRABP1, PARP1, KAT2B, PRKCI, MAPK14, PRKAR2B, TAF4, ERCC3, IGFBP3, NCOA1, PRKAG2, **PRKCH**, PRKCB	
	91	0.269	0.022	PRKAR2B, MED1, ADCY3, RDH13	

^1^pathways are shown that were significant at p < 0.05 according to Fishers exact test in at minimum one of the three types of comparisons.

^2^ratio of number of differentially expression genes assigned to the pathway and the total number of genes assigned to the pathway in the Ingenuity Knowledge Base.

## Discussion

### Differential expression at bud formation and growth among divergent breeds

The mammary gland development begins during fetal development and proceeds through the adult life. The fetal mammary gland development in pigs initiates at day 23 (E23) with the formation of the milk lines and continues with the invagination of epithelial cells to form buds that subsequently increase in size accompanied by the condensation of surrounding mesenchymal cells. The mammary gland development at fetal stages depends on the signal between epithelium and surrounded mesenchyme. Epithelial and mesenchyme are found in every organ. Either the transitions of epithelial into mesenchyme or the transitions of mesenchyme into epithelial is the cause of morphogenesis, differentiation and growth during the development [14]. A mesenchymal-epithelial interaction is a reciprocal process, where mesenchyme induces the formation of epithelial, while epithelium induces mesenchymal differentiation [15,16]. In fetal mammary gland development, mammary mesenchyme induces the formation of mammary epithelial buds when they were combined with the epidermis. The mammary buds induce the expression of androgen receptors (AR) and tenascin in mesenchymal cells and then the formation of the primary mammary mesenchyme occurs [15]. The insufficient proliferation of mesenchyme in embryonic mammary development may cause the inverted teat [13].We analysed samples 63 dpc and 91 dpc during growth of buds and their protrusion by mesenchymal cell development that is important for the postnatal shape and function of teats. Furthermore, hypothesising that the prenatal development of the buds affects the postnatal phenotype of teats we compared samples of two breeds that are subject to different selection pressure on number, shape and function of teats. The analysis of differential expression in the fetal tissues at the two time points between the two breeds showed the largest distinction of samples of the breed GL at 63 dpc from all other samples. Accordingly, the identification of transcripts with different abundance in specimens of GL at 63 dpc compared to those of PI at the same developmental stage or compared to that of GL of a later stages (91 dpc) at p < 0.05 was achieved with considerable low false discovery rates (q ≤ 0.03 and q ≤ 0.14, respectively). Together with the relatively large differences between 63 dpc and 91 dpc in GL and moderate divergence between early and late PI samples as well as between GL 91 dpc and PI 91 dpc this indicates that in GL at 63 dpc a less progressed status has been reached and catching up on the development proceeds until 91 dpc. The assignment of DE-genes to biofunctions and canonical pathways underline the relatively delayed development in GL compared to PI in terms of differentiation, maintenance, and maturation and a thus prolonged proliferative phase.

### DE genes essentially belong to signaling pathways

The development of embryonic mammary gland can be divided into several steps as described above. Many known genes were regulated in each stage of mammary development. These genes were assignment to signaling pathways whereas expression of genes of metabolic pathways was not differentially regulated among the samples. Previous studies have already addressed a number of genes of signaling pathways in particular those related to growth factors. Fibroblast growth factor 2 (FGF2) and its receptor (FGFR2) were found differentially expressed in this study (Table 2). Fibroblast growth factor (FGFs) together with T-box transcription factor were found in mammary line in early development and members of the Wnt gene family were also found in mammary placodes. Members of the Wnt signaling pathway were differentially expressed between 63 and 91 dpc. They play a role in the formation of placodes and mammary epithelial differentiation [17-19]. Lymphocyte enhancer factor-1 (LEF1), an important factor for the mammary placodes and bud maintenance, is a transcription factor, which is defined in epithelial cell of mammary buds and mesenchyme [20]. It was found regulated due to breed and stage in this study (Tables 1 and 2). The expression of LEF1 is induced by parathyroid hormone - protein (PTHLP) and its receptor (PTHR1) [21]. PTHLH (parathyroid hormone like hormone) and PTHR1 genes were clearly detected in epithelial and connective teat tissues. Polymorphisms of PTHLH and PTHR1 as well as LEF1 were associated with the inverted teat phenotype [22,23]. Studies of mice deficient of FGFs, Wnts, LEF1 and PTHLH show that these are required for adequate signaling in fetal mammary development [24-28].

The development of fetal mammary gland largely depends on interaction between mesenchymal-epithelial. The basic molecules for signals in mesenchyme-epithelial interaction are provided by paracrine signaling, which consists of epithelial tyrosine kinase receptors and their mesenchymal ligands [29]. Fibroblast growth factor (FGF) signaling that was addressed in this study, like Hepatocyte growth factor (HGF) signaling, Growth hormone signaling, and Epidermal growth factor (EGF) signaling are the main cellular growth, proliferation and development signaling pathways. Accordingly, genes (GAB1, MAPK9, PIK3C2B, PIK3C3 and PRKCH) from signaling pathways were selected to validate microarray data that all have known roles in developmental processes.

GRB2-associated binding protein 1 (GAB1) is a member of the family of docking proteins. GAB1 functions as a mediator in growth factor signaling and cytokine receptor, especially in EGF signaling, HGF signaling and Platelet-derived growth factor (PDGF) signaling which stimulates the migration of epithelial, mesenchymal and hematopoietic cells during embryogenesis and branching morphogenesis of epithelial cells. The deficiency of GAB1 results in the lack of ligands, receptors and signaling molecules and develop the organs [30-34].

Mitogen-activated protein kinase 9 (MAPK9), which is known as c-Jun NH_2_-terminal kinase 2 (JNK2), is a member of the MAP kinase family. It also enhances in the signal transduction pathways. MAPKs are regulated through cascades composed of MAPK, MAPK kinase (MAPKK, MKK or MEK) and MAPKK kinase or MEK kinase (MAPKKK or MEKK). These kinases are activated through hormones and growth factors that act via RTKs (e.g. EGF, PDGF and FGF) or cytokine receptors (e.g. growth hormone). MAPKs function in gene transcription, protein synthesis, cell cycle, cell death, and cell differentiation. MAPKs may involve in epithelial-mesenchymal transition (EMT). The overexpression of MAPK induced cell migration and invasion, a morphologic change in EMT. The lack of MAPK increasing tumor aneuploidy and reduced DNA damage [35-40].

Phosphoinositide-3-kinase, class 3 (PIK3C3) and phosphoinositide-3-kinase, class 2, beta polypeptide (PIK3C2B) belong to the phophoinositide-3-kinase (PI3K) family. PI3Ks are a family of unique and conserve intracellular lipid kinases that are activated by growth hormones, RTKs and G-protein-coupled receptors (GPCRs). PI3Ks are capable of phosphorylation the 3´-hydroxyl group of phosphatidylinositol 4,5 diphosphate (PIP_2_) to generate phosphatidylinositol-3,4,5-trisphosphate (PIP_3_), which activates intracellular signaling pathways for regulated function in cell growth and proliferation, cell migration, and cell metabolism. PI3Ks can induce the differentiation of cells of epithelial or mesenchymal origin. During EMT, TGFß signaling co-operates with Rho signaling to activate PI3K. PRL-3 signals through PI3K and then leads to EMT [41-45]. PIK3C3 homozygous mutant causes cell proliferation defect, embryonic lethal and death [46].

Protein kinase C, eta (PRKCH) is calcium independent and phospholipids dependent member of the protein kinase C (PKC) family. PKC, a family of serine and threonine - specific protein kinases, has important roles in cell proliferation, survival, migration and adhesion. PKC is activated via lipid activators and phosphorylation. PKC are classified into α, ß_I_, ß_II_, γ, δ, ϵ, η (L), θ, μ, ξ, λ isoforms. PKC is predominantly a cytosolic enzyme in the mammary gland and have a role in prolactin (PRL) to stimulate lactogenic processes. PKC- α isoform has a role in mammary cell differentiation, while PKC- δ isoform is mediated by MEK/ERK pathway and induce the production of proteolytic enzymes [47-52].

## Conclusions

The study provides a holistic view on the gene expression during prenatal development addressing the abundance of 11,731 probe sets at two fetal stages at the phase of growth and protrusion of mammary buds in two pig breeds. It is shown that the development in PI precedes GL in a way that processes of differentiation, maintenance, and maturation are predominant whereas in GL still proliferative processes are prevalent. These differences in fetal development have implications on the postnatal phenotypes. Due to divergent selection pressure, GL and PI differ in the number of teats and in functional morphological criteria of mammary complex quality, like position and symmetry, shape, and functionality. In particular there is large divergence in the prevalence of inverted teats, a disorder characterized by the formation of non-functional teats. We hypothesed that differential expression between the two breeds at fetal stages reflect the prenatal initiation of postnatal phenotypes also in terms of the liability to emerge inverted teats. We have previously identified molecular routes that are differentially regulated in normal compared to inverted teats at peripubertal stages [11,13]. We questioned here, whether these molecular routes are already relevant at prenatal stages and thus their modulation may initiate the postnatal development. In fact, the DE-genes were mainly from signaling pathways that are known to have implication for mammary gland development. The finding of overwhelming importance of signaling pathways in teat development and of differential expression of related genes, which suggest a prolonged proliferative development in the breed GL selected against undesired teat phenotypes and which potentially promotes the emergence of inverted teats, is in line with our previous findings when comparing normal and inverted teats at postnatal stages [13].

## Materials and methods

### Sample collection and RNA preparation

Animal care and tissue collection processes followed the guidelines of the German Law of Animal Protection, and the experimental protocol was approved by the Animal Care Committee of the Leibniz Institute for Farm Animal Biology (FBN, Dummerstorf, Germany). The milk lines samples were collected from14 fetuses of sows of the breed German Landrace (GL) and the Pietrain (PI) at the 63 and 91 days post conception (dpc) (63 dpc GL n = 4; 91 dpc GL n = 4; 63 dpc PI n = 3; 91 dpc PI n = 3, respectively). After collected, the samples were immediately frozen in liquid nitrogen and stored at −80°C.

For isolation of total RNA, nipples were prepared from the milk line tissues with scalpel and grinded in a mortar under liquid nitrogen. Then they were homogenized with 1 ml Trizol Reagent (Sigma-Aldrich, Taufkirchen, Germany) by using syringes and needles and cleaned up with the NucleoSpin RNA II kit (Macherey-Nagel, Düren, Germany). In addition, the DNAse treatment was done according to the manufacturer’s protocol. After that, the integrity of the RNA samples were checked by visualizing them on 1.5% agarose gel contained formaldehyde stained with ethidium bromide. The concentration levels of RNA were measured by a Nano Drop ND-1000 Spectrophotometer (PEQLAB, Erlangen, Germany). The absence of DNA contamination was checked by using the RNA as a template in PCR amplifying fragments of the glyceraldehydes-3- phosphate dehydrogenase (GAPDH) gene. All RNAs were stored at −80°C for further analysis.

### Microarray analysis

Affymetrix GeneChip Porcine Genome Arrays (Affymetrix, St. Clara, USA) containing 24,123 probe sets were used in the expression study. A total 14 RNA samples from different breeds and stages were used for the array hybridization. cDNA was synthesized from total RNA and used to generate biotin-labeled cRNAs target according to the Affymetrix protocol. The biotinylated cRNA was fragmented and used for hybridization to the Affymetrix Gene Chips at 45°C for 16 hours. Hybridization, washing, and scanning of the arrays were done according to the manufacturer’s recommendation. The data were analyzed with the Affymetrix GCOS 1.1.1 software by using the global scaling to a target signal of 500.

### Statistical analysis

The raw intensity files (*.cel) were used in microarray data analysis with the Affymetrix Expression Console software (Affymetrix, St. Clara, USA). First the data were processed with the MAS5.0 algorithm to generate probe cell intensity values, i.e. single expression value for each probe set that are derived from intensities of pairs of perfect-match probes and mismatch probes, and to evaluate presence and absence of transcripts. Using default settings (detection p-values of <0.04 for `present´, ≥0.04 and ≤0.06 for `marginal´, and <0.06 for `absent´) only `present´ calls were used. The subsequent data processing, including background correction, probe summarization and normalization, was performed using the probe logarithmic intensity error (PLIER) algorithm that reveal summary values for the probe sets (Affymetrix 2001, 2005). The microarray data related to all samples were deposited in the Gene Expression Omnibus public repository (GEO accession number: GSE32956). The probe intensity value obtained by the PLIER algorithm were transformed to logarithms and evaluated by analysis of variance taking into account the effects of stage and breed and their interaction to detect genes differentially regulated at p < 0.05 (JMP Genomics). Corresponding q-values were calculated according to algorithms established by Storey [53] using QVALUE. Based on BLAST comparison of the Affymetrix porcine target sequences with the porcine genome sequence (Ensembl_Sscrofa_9), 20,689 of the 24,123 probe sets on the Affymetrix Porcine GeneChip were annotated [54]. This source of annotation was used in this study for Ingenuity Pathways Analysis (IPA) (Ingenuity Systems, http://www.ingenuity.com). The Ingenuity applications were used to generate networks and assess statistically relevant biofunctions and canonical pathways associated with the microarray data. The significance of the association between the dataset of differentially expressed genes (DE-genes) and the predefined pathways and functional categories of the Ingenuity Knowledge Base was measured by Fischer’s exact test and adjusted using the Benjamini–Hochberg correction, providing a p-value, which would determine the probability that the association between the genes in the dataset and the pathway is explained by chance alone.

### Quantitative real time PCR

1 μg of total RNA of the same individual samples used for microarray analysis were reverse transcribed with Super Script III with Oligo (dT) and random primers (Invitrogen, Karlsruhe, Germany) in a total volume of 10 μl. The cDNAs were used as the template for validation of the gene expression levels by quantitative RT-PCR in duplicate. Quantitative RT-PCR was conducted with the LightCycler 480 system (Roche, Mannheim, Germany). The reaction volume of 10 μl contained 5.0 μl of LightCycler 480 SYBR Green I Master (Roche), 600 nM of each primer, and 2 μl of cDNA. Amplification conditions were 95°C for 10 min, 40 cycles of 95°C for 15 sec, 60°C (annealing) for 10 sec and 72°C for 15 sec. The primers were designed from Affymetrix core sequence with Primer3 (http://frodo.wi.mit.edu/primer3). The list of primer sequences is provided in Table S1 (Additional file 2). Standard curves were derived for each gene from a serial dilution of cDNAs. For all the assays threshold cycles were converted to copy numbers using the standard curves generated by amplifying serial dilutions of an external PCR standard (10^7^ - 10^2^ copies). At the completion of the amplification protocol, all samples were subjected to melting curve analyses and gel electrophoresis to verify the absence of any non-specific product. Copy numbers of the house keeping genes HPRT1 and RPL32 were obtained from each individual sample to enable accounting for variation in RNA input and efficiency of reverse transcription by normalization when calculating mRNA copy numbers of the target genes. Both of the reference genes were checked for lack of variation on the basis of microarray data at prenatal and postnatal stages [13,55]. Data were analyzed like microarray data by analysis of variance including the effects of stage and breed (JMP Genomics). Differences were considered significant at p < 0.05.

## Authors’ contributions

Conceived and designed the experiments: SP, EM, KW. Performed the experiments: KC. Analyzed the data: KC, EM, SP, KW. Contributed reagents/materials/analysis tools: SP, EM, KW. Wrote the paper: KC, KW. All authors read and approved the final manuscript.

## Supplementary Material

Additional file 1 **Figure S1.** Significant biofunctions (top 20 according to p-value) representing genes differentially expressed between 63 dpc and 91 dpc in Pietrain and German Landrace. All assignments significant after Benjamini–Hochberg correction. Click here for file

Additional file 2 **Table S1.** List of genes and corresponding primers used for quantitative RT-PCR. ^1^Reference genes. Click here for file
