# Supplementary figures and images for: Gene expression analysis of mammary tissue during fetal bud formation and growth in two pig breeds – indications of prenatal initiation of postnatal phenotypic differences
Source: BMC Dev Biol. 2012 Apr 26;12:13. doi: 10.1186/1471-213X-12-13 (PMC3527354; doi:10.1186/1471-213X-12-13)

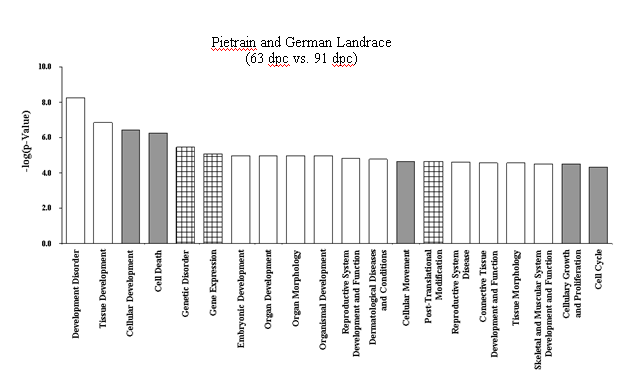

Supplement: Additional file 1 — Figure S1. Significant biofunctions (top 20 according to p-value) representing genes differentially expressed between 63 dpc and 91 dpc in Pietrain and German Landrace. All assignments significant after Benjamini–Hochberg correction. [file 1471-213X-12-13-S1.tiff]
