# Supplementary material for: Gene expression analysis of mammary tissue during fetal bud formation and growth in two pig breeds – indications of prenatal initiation of postnatal phenotypic differences
Source: BMC Dev Biol. 2012 Apr 26;12:13. doi: 10.1186/1471-213X-12-13 (PMC3527354; doi:10.1186/1471-213X-12-13)
Supplement: Additional file 2 — Table S1. List of genes and corresponding primers used for quantitative RT-PCR. 1Reference genes. [file 1471-213X-12-13-S2.doc]

**Table S1:** List of genes and corresponding primers used for quantitative RT-PCR.

| **Gene symbol** | **Gene name** |  | **Sequence 5´-3´** |
| --- | --- | --- | --- |
| **GAB1** | GRB2-associated binding protein 1 | for | actgccccaaataccgtaaa |
|  |  | rev | ttgtggtcttgacagccttg |
| **MAPK9** | Mitogen-activated protein kinase 9 | for | accccttgaaggttgtcgat |
|  |  | rev | acaactgagtaggggcaagg |
| **PIK3C2B** | Phosphoinositide-3-kinase, class 2, beta polypeptide | for | gggatgccctaactgaacaa |
|  |  | rev | aatccatccggtagggaaag |
| **PIK3C3** | Phosphoinositide-3-kinase, class 3 | for | aaagcctcttcctcctccaa |
|  |  | rev | ccaccatcaaggaaaacagg |
| **PRKCH** | Protein kinase C, eta | for | tgccacaatacccagtagca |
|  |  | rev | tgagctgcctaatgggagaa |
| **HPRT^1^** | Hypoxanthine phosphoribosyltransferase | for | gtgatagatccattcctatgactgtaga |
|  |  | rev | tgagagatcatctccaccaattactt |
| **RPL32^1^** | Homo sapiens ribosomal protein L32 | for | agcccaagatcgtcaaaag |
|  |  | rev | tgttgctcccataaccaatg |

^1^Reference genes.
